# Large Suspended Monolayer and Bilayer Graphene Membranes with Diameter up to 750 µm

**DOI:** 10.1038/s41598-020-63562-y

**Published:** 2020-04-14

**Authors:** Shirin Afyouni Akbari, Vahid Ghafarinia, Tom Larsen, Marsha M. Parmar, Luis Guillermo Villanueva

**Affiliations:** 1 Isfahan University of Technology (IUT), Isfahan, Iran; 2 Advanced NEMS Laboratory, École Polytechnique Fédérale de Lausanne (EPFL), Lausanne, Switzerland

**Keywords:** Engineering, Nanoscience and technology

## Abstract

In this paper ultra clean monolayer and bilayer Chemical Vapor Deposited (CVD) graphene membranes with diameters up to 500 µm and 750 µm, respectively have been fabricated using Inverted Floating Method (IFM) followed by thermal annealing in vacuum. The yield decreases with size but we show the importance of choosing a good graphene raw material. Dynamic mechanical properties of the membranes at room temperature in different diameters are measured before and after annealing. The quality factor ranges from 200 to 2000 and shows no clear dependence on the size. The resonance frequency is inversely proportional to the diameter of the membranes. We observe a reduction of the effective intrinsic stress in the graphene, as well as of the relative error in the determination of said stress after thermal annealing. These measurements show that it is possible to produce graphene membranes with reproducible and excellent mechanical properties.

## Introduction

Graphene films have raised lot of attention in the past 15 years due to their outstanding material properties. In particular, given the combination of mechanical and electrical properties, these films are very promising for micro- and nano-electromechanical systems (MEMS & NEMS) and their applications^1–14^. Most of the studies on graphene are usually performed on supporting substrates such as SiO_2_/Si. However, substrate induced carrier scattering, dopants and phonon leakage obscure the intrinsic properties of graphene significantly. Suspended graphene has revealed superior properties in certain aspects, and thus it becomes an ultimate platform to exploit the capabilities of the material^1,2,15^. From a mechanical standpoint, since graphene is formed by strong sp^2^ covalent carbon-carbon bonds, it presents the largest Young’s modulus in nature (together with carbon nanotubes) with 1 TPa^1^. In addition, when very small areas are probed, it behaves extremely close to an ideally brittle solid, showing an incredibly high yield strength^1^. But this is when we can probe a single grain, with no dislocation. Polycrystalline graphene, on the other hand, contains randomly distributed topological defects such as dislocations and grain boundaries^16–18^. These defects affect the effective properties measured in the devices, both electrically^19^, mechanically^20^, and thermally^21^. In particular, it has been shown that grain boundaries reduce graphene strength substantially, even with small misorientation angles^22^. Therefore, despite of its ultrahigh theoretical strength, polycrystallinity threatens the practical applications of graphene as a mechanical material.

There are many applications for which a suspended atomically thin membrane (of e.g. graphene) has been proposed to reach the ultimate performance. These include desalination^23^, DNA sequencing^24,25^, pressure sensors^26^, temperature sensors^27^, gas sensors^28^, bolometers^29^. For most of these applications, having extreme aspect ratio membranes, i.e. a large area and extremely small thickness (few monolayers); and free of residues would be extremely beneficial. However, that also means very fragile structures from a mechanical standpoint and an increasing difficulty to obtain large aspect ratio membranes.

Different techniques for fabricating graphene membranes have been reported, such as patterning and electroplating^30^, direct etching of a metal catalyst^31^, critical point drying to avoid the drag force from surface tension^32^ and wet transferring^33–35^. The latter is the most popular method to fabricate stand-alone membranes of various aspect ratios. However, the membranes are frequently ruptured because of the surface tension when the rinsing liquid is evaporating and, consequently, the size and yield are limited. Typical sizes of monolayer graphene membranes on a perforated substrate are in the order of tens of micrometers^17,18,36^. In 2014, Lee *et al*., published a strategy called an inverted floating method (IFM)^35^. With this strategy, the sample is rinsed without being immersed in liquid, but rather letting the sample float on the surface of an acetone bath. This prevents the liquid entering into the holes underneath the graphene membrane and increases the yield of large membranes, providing suspended monolayer graphene membranes with diameters up to ∼ 500 *μm*^35^, but it suffers from large amounts of polymer residue on the graphene surface^37^. Therefore, development of a large scale, free-standing, and clean graphene membrane is still a big challenge.

In this paper, we used IFM followed by thermal annealing in vacuum for fabricating ultra clean monolayer and bilayer chemical vapor deposited (CVD) graphene membranes with diameters up to 750 µm. These membranes are free of cracks, wrinkles and pinholes. Also, the graphene membranes are very clean with almost no PMMA residuals. SEM and optical profilometry images confirm our claim. Furthermore, the resonating properties of the membranes with different diameters are characterized by Laser Doppler Vibrometer (LDV) before and after thermal annealing. The results show that the thermal annealing removes PMMA residuals on the graphene membranes significantly.

## Fabrication of CVD graphene membranes

**Figure 1:**
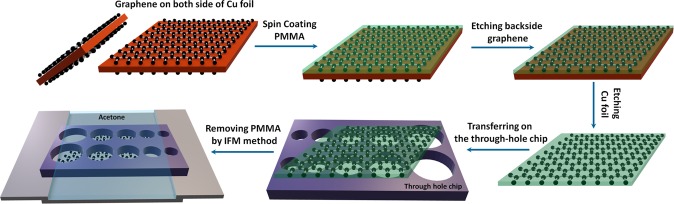
CVD Graphene transferring proccess. The schematic of the transferring procedure for fabricating large suspended graphene membranes. Suspended CVD graphene was fabricated using typical procedures, including polymethyl methacrylate (PMMA) spin-coating, backside graphene etching, copper foil etching, rinsing, and wet-transfer onto a perforated SiO_2_/Si substrate. After the PMMA/graphene membrane was thoroughly dried, PMMA was removed using the Inverted Floating Method.

**Figure 2:**
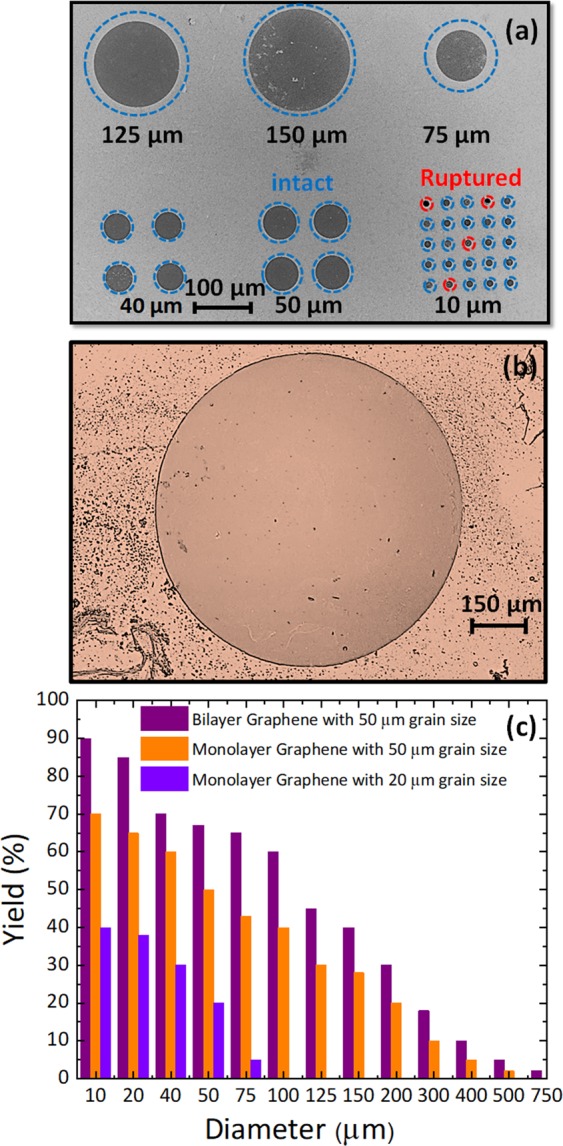
Membranes after removing PMMA using IFM. (**a**) SEM image of bilayer graphene membranes with different diameters. Intact and ruptured graphene membranes are respectively rounded by blue and red dashed circumferences. (**b**) Optical image of a 750 *μm* in diameter bilayer graphene membrane. (**c**) Yield of membranes made out of Monolayer Graphene with small grain sizes (<20 *μm*); and Monolayer and Bilayer graphene with big grain sizes (> 50 *μm*). The effect of the grain size is evident, as it also is the effect of having a second atomic layer in the device.

To fabricate the membranes, we purchase CVD graphene and transfer it onto Si chips with predefined through-holes chips having diameters ranging from 10 *μm* to 1000 *μm*^35,37^. Figure 1 depicts a schematic of the transfer process we use, involving a PMMA removal by IFM. Briefly, a layer of 150 nm thick PMMA is coated onto the CVD graphene which has been grown on copper foil. For the purpose of comparison, we show here results obtained for three different CVD graphene, from two vendors. After etching the backside graphene with oxygen plasma, the copper foil is etched in ammonium persulfate. To rinse the graphene after copper etching, the PMMA/graphene stack is left in de-ionized water for 24 hours. Then the stack is transferred onto a Si chip with through-holes and a 300 nm thick layer of SiO_2_. After wet transferring, the PMMA/graphene stack remains well suspended over the holes without rupturing, even in the case of 1 mm holes. We then leave the chip for 24 hours in a clean and dry environment in order to evaporate the water between the graphene and the perforated substrate. During this period, the water surface tension drags the film into contact with the substrate. Unfortunately, this also breaks some of the membranes. Finally, PMMA is carefully removed by exposing the PMMA-covered side of the chip to acetone, but avoiding the solvent to go into the holes (IFM)^35^. To do this, we use a custom-made stand (see Fig. 1). Acetone is then pumped very slowly during 2–120 min. We do not observe much change in the residues between leaving the chip for 2 and 120 mins in acetone, but we observe a decrease in the yield of the larger membranes when leaving the chip for longer times in acetone. After removing the PMMA, the sample is quickly placed in a vertical position to minimize residues on the graphene surface. After finalizing the transfer, we inspection the chips using optical and scanning electron microscopes. First, we use this inspection to establish the yield of the process as a function of the membrane diameter, which we define as the ratio of membranes before and after removing PMMA. Typical inspections can be seen in Fig. 2a and Fig. 2b. In Fig. 2a, intact and ruptured graphene membranes are respectively rounded by blue and red dashed circumferences.

As mentioned above, we explored three different types of graphene: CVD monolayer with average grain size of 20 µm (vendor #1), CVD monolayer with average grain size of 50 µm (vendor #2) and CVD bilayer with average grain size of 50 µm (vendor #2). The survival yield for each of the three types is shown in Fig. 2c, and it shows, as expected, a decrease with the hole diameter. Importantly, it also shows the effect of the type of graphene used in the transfer. Increasing the grain size from 20 µm to 50 µm allowed us to have membranes 7 times larger (monolayer). The use of bilayer graphene also increases the yield substantially, but the effect is less important than the grain size. In fact, this evidences our very first conclusion within this work: the choice of a proper graphene vendor can really affect the outcome of your fabrication process and your yield. This is in line with what has been noticed by other groups in the case of graphene flakes^38,39^. In our case, it is mostly related with the grain size, as it has been reported by several sources and both by atomistic simulations^40^ and by experimental results^41–43^, that the grain boundaries reduce the yield strength of the material.

**Figure 3:**
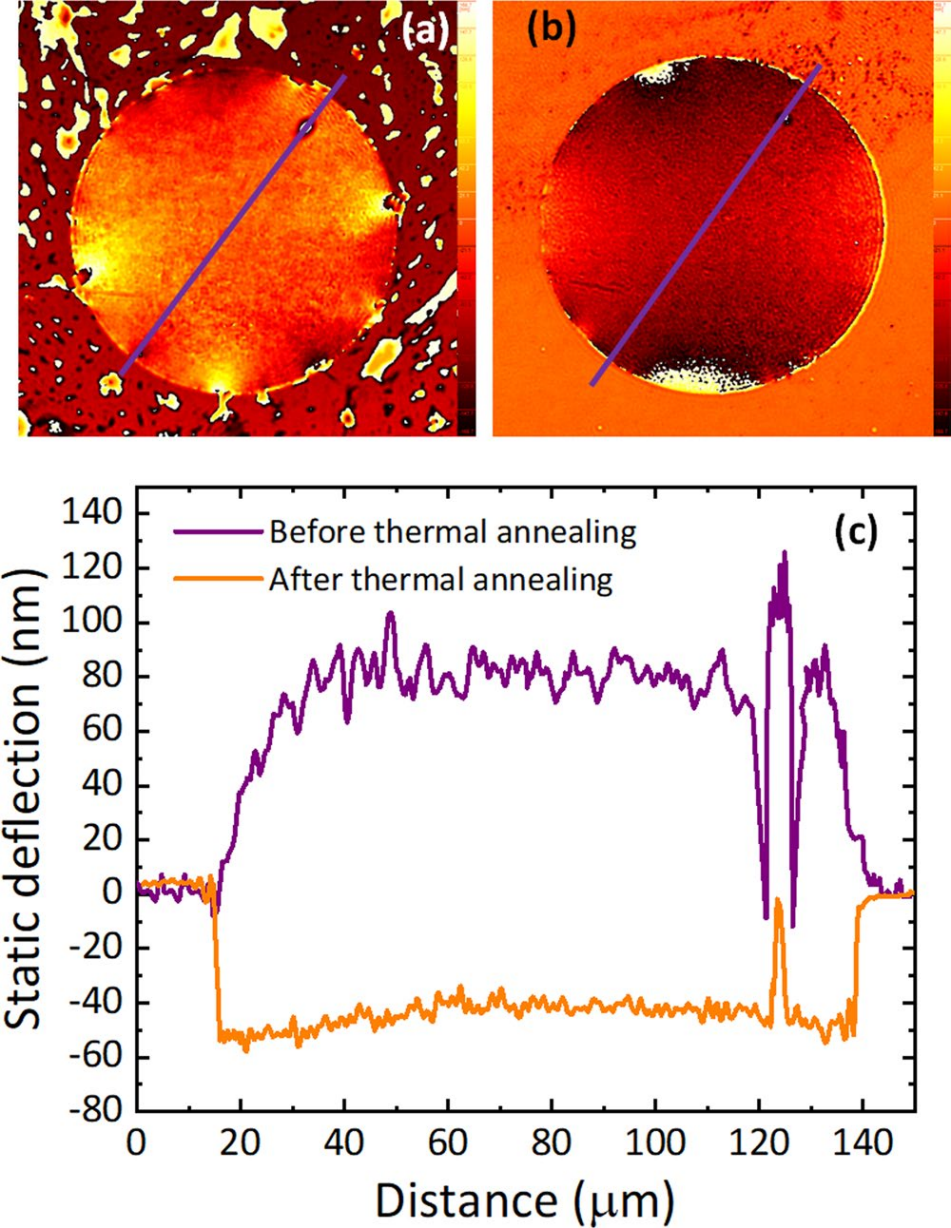
Effect of thermal annealing. Phase images of 125 *μm* in diameter bilayer graphene membrane obtain by 3D optical profilometry (**a**) before thermal annealing. (**b**) After thermal annealing. (**c**) Static deflection of the 125 *μm* in diameter bilayer graphene membrane along the purple line before and after thermal annealing. Static deflection is less noisy after thermal annealing and it shows that the amount of PMMA residuals decrease significantly.

In addition to grain boundaries, other defects coming from the growth, such as silica particles, metal residues, etc. can also reduce the yield of the membranes. Alternatively, and together with PMMA residues, they can remain on the membranes after release, as it can be seen in Fig. 2a,b, and Fig. 3. To further remove PMMA residues after release, we perform a two hours thermal annealing at 250 °C in vacuum (10^−4^ mbar). The effect of this annealing is illustrated in Fig. 3. By comparing Fig. 3.a and Fig. 3b (both images taken with a Digital Holographic Microscope from LyncéeTec^44,45^) we can see how the surface becomes much cleaner (from 7 nm to 2.5 nm RMS), except for a large defect that we assume it to be a silica residue from the growth. In addition, we can also see how the membrane after annealing changes its conformality on the hole region, as we find a step of around 50 nm when the hole starts. During this annealing process, unfortunately, some of the membranes break (especially for larger diameter). The main reason for this is the thermal expansion coefficient mismatch between the graphene and the substrate, since we observe a larger percentage of ruptured membranes when we anneal at higher temperatures.

## Characterization

**Figure 4:**
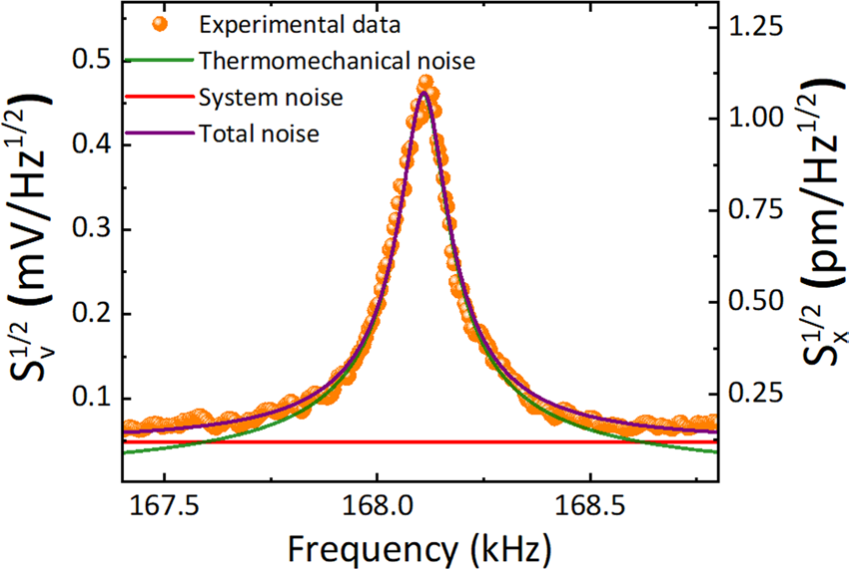
Thermomechanical noise measurement of a graphene membrane. By performing a nonlinear fitting to a Lorentzian with background, we can extract the resonance frequency and quality factor. This particular case corresponds to a monolayer graphene membrane with 200 µm diameter (orange scattered data), with resonance frequency of 168.1 kHz and quality factor of 1580.

We then characterize the dynamical behavior of the fabricated membranes, by measuring their thermomechanical noise, using an LDV OFV-551 from Polytec^45^, which is then fitted to a Lorentzian curve to extract both resonance frequency and quality factor^46^. Figure 4 shows a typical result, in particular for a monolayer graphene membrane of 200 µm in diameter with resonance frequency of 168.1 kHz and a quality factor of 1580 (in vacuum, at ${10}^{-5}$ mbar).

**Figure 5:**
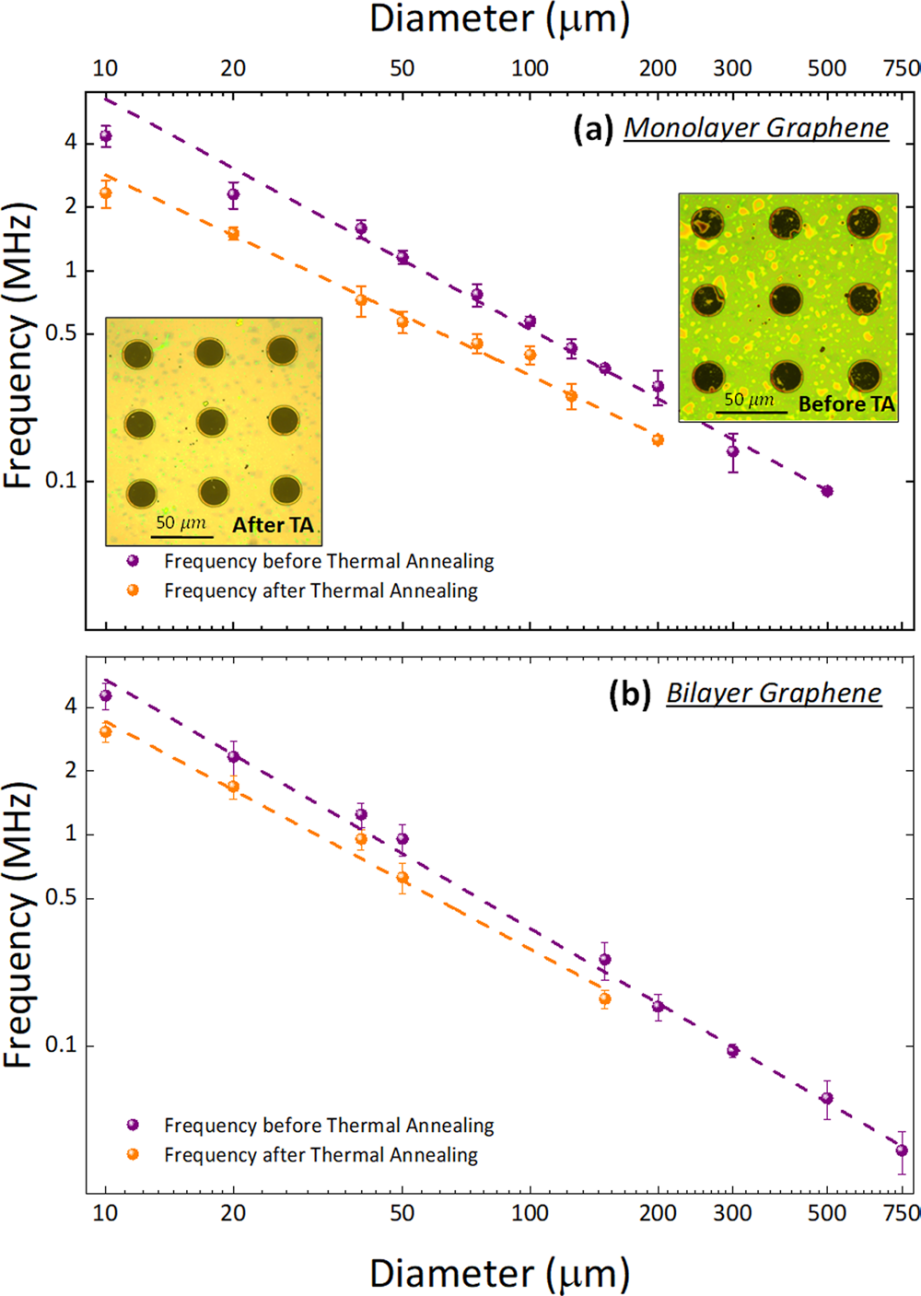
Measured resonance frequency. Resonance frequency as a function of membrane diameter before and after thermal annealing in vacuum (10^−4^ mbar) for 2 hours at 250 °C. (**a**) Monolayer graphene membranes. Inset: (i) Before thermal annealing. (ii) After thermal annealing. (**b**) Bilayer graphene membranes. The purple and orange dashed lines are fits that reveal ${f}_{r}\sim {D}^{-1}$ for all the membranes before and after thermal annealing. Since during thermal annealing a big part of polymer residues are removed, this helps us better determine the intrinsic stress present in the membranes.

We first measure the power spectral density (PSD) of the signal in volts, and convert it to amplitude at the center of the membrane by using the method described in^46^, to obtain for example a responsivity of 43 nm/V in the case of Fig. 4. Using thermomechanical noise, we estimate the frequency and quality factor of more than 150 devices of different dimensions. Figure 5 summarizes the results for the resonance frequency and we separate monolayer (a) and bilayer (b) devices with big grain sizes (since the samples with small grain size did not offer a high-enough yield at large diameters). The dependence of the frequency vs the diameter follows closely the prediction for a membrane^46^, with a ${D}^{-1}$ dependence. Indeed, the full expression for the frequency of a graphene membrane is given by:

1 $$f=\frac{4.808}{2\pi D}\sqrt{\frac{{\sigma }_{s}}{{\rho }_{s}}}$$

where *D* is the diameter of the membrane, ${\rho }_{s}$ is the surface mass density of graphene and ${\sigma }_{s}$ is the surface stress. In principle, if the membranes were made of pristine graphene, one could use the experimental results for the frequency to determine the stress level in our graphene samples. However, due to the amount of residues on top of the graphene after transfer, this estimation is not accurate. It is then that we perform the thermal annealing in vacuum described in the previous section and, without breaking the vacuum, we measure again the thermomechanical noise of every membrane that remains alive (some of the bigger membranes break during annealing) and extract the resonance frequency and quality factor. The results can also be seen in Fig. 5, in orange, for mono- and bilayer graphene.

The equivalent surface stress that we obtain from the experimental data before annealing is: $1.7\pm 0.3{\rm{m}}\,{\rm{N}}/{\rm{m}}$ and $3.4\pm 0.3\,{\rm{mN}}/{\rm{m}}$ for mono- and bi-layer respectively, assuming a surface density of ${\rho }_{s}=680\cdot {10}^{-9}\,{\rm{kg}}/{{\rm{m}}}^{2}$. We believe that the difference in estimated stress between both samples (and its dispersion within each sample) is large mostly because of the residues, which are very different in every sample. On the other hand, after annealing we estimate the stress to be $1.2\pm 0.15\,{\rm{mN}}/{\rm{m}}$ for both mono- and bilayer. Thermal annealing affects the resonators in two ways: (i) it relaxes the graphene sheets due to the thermal cycling, and (ii) it removes the residues. The latter point makes all membranes close to the pristine case, therefore the dispersion is much smaller and the estimation is more accurate, since the mass-loading effect of the residues disappears. In fact, the difference between mono and bilayer after annealing is within the standard deviation. In addition, the stress is reduced because the thermal cycling relaxes the membranes, which causes the resonance frequencies to reduce even after the loss of mass.

**Figure 6:**
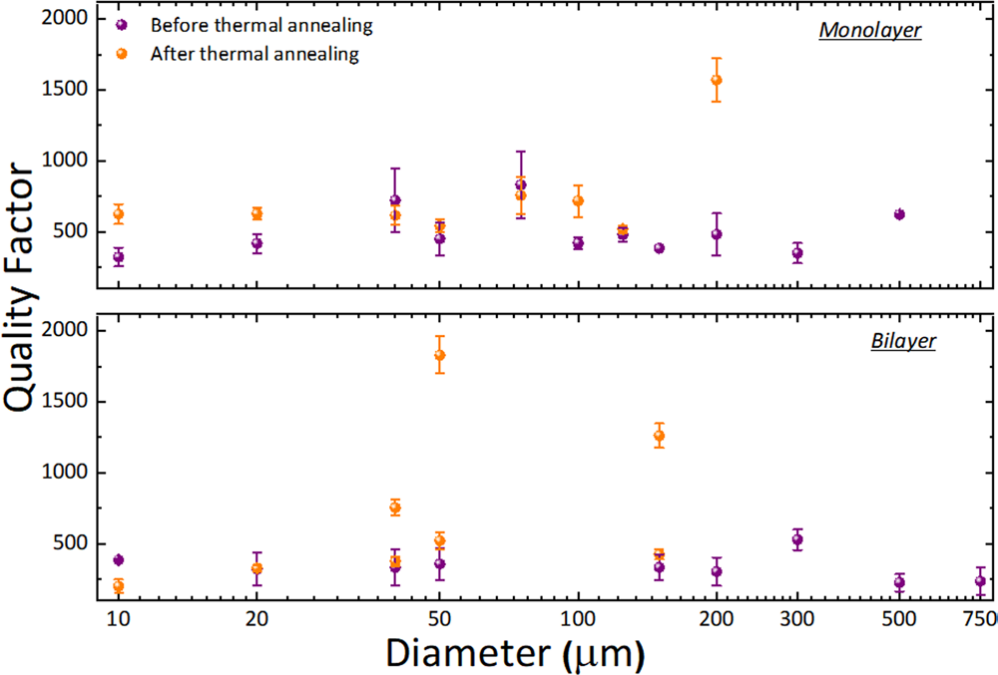
Measured Quality factor. Quality factor of monolayer and bilayer graphene membranes as a function of diameter before and after thermal annealing. Typically, quality factor increased, and standard deviation of quality factor decreased after thermal annealing but also in some case quality factor decreased.

We can also compare the quality factor (*Q*) extracted from our measurements for the devices before and after thermal annealing, which can be seen in Fig. 6. Contrarily to what was reported by Barton *et al*^47^., we do not see an increase of our quality factor with increasing diameter. This might be because we are mostly probing on another range of diameters (from 10 µm up to 750 µm compared to 2.5µm-25µm in^47^), or because we have a different main damping mechanism.

After annealing, i.e. after removing most of the polymer residues, one could expect an increase of the quality factor, since polymer residues present very large internal energy losses. However, the change in *Q* was not consistent across our devices. In some cases, the increase was much larger than in others and, importantly, in some rare cases we also observed a reduction in *Q* after thermal annealing. We understand this as evidence that our quality factor is not determined by the energy losses associated to the intrinsic losses in the polymer residues. In that case, even though we reduce the damping associated with the polymer residues on top by removing said residues, there are other effects that might remain constant or even increase with the annealing. For example, we could be forming small cracks on the membranes during the annealing, thus increasing the losses. The stress is also reduced, as we have discussed above, thus reducing the energy stored in our resonators and consequently reducing *Q*^48^. Finally, it can also be due to a change in the clamping conditions, since it has been shown that clamping can notably influence *Q*^49^, and we see that after annealing the clamping conditions in our devices change (see Fig. 3).

## Conclusions

We demonstrate the transfer of monolayer and bilayer CVD Graphene with small and big grain domains on through-hole chips with diameters ranging from 10 µm to 750 µm by a PMMA-mediated approach. The PMMA layer is removed by IFM followed by thermal annealing for 2 hours at 250 °C in vacuum (10^−4^ mbar). This yields ultra clean, monolayer and bilayer graphene membranes with diameters up to 500 µm and 750 µm, respectively, but only if the grain size is large, not for sample with smaller grains. Dynamic mechanical properties of fabricated devices are characterized via interferometric detection of their thermomechanical noise. The resonance frequencies are inversely proportional to the diameter of the membranes, in accordance with theory. After cleaning, we observe a reduction of the effective stress as well as a reduction of its relative dispersion, showing that it is necessary to clean the resonators before estimating the intrinsic stress. We believe our findings will help in the development of different types of mechanical sensors that benefit of very large aspect ratio to optimize their performance.

## Methods

### Transferring Graphene on a hole-through chip

A 150 nm-thick PMMA (e-beam resist, 495 k A2, Microchem) was spin-coated on the monolayer and bilayer CVD graphene on copper foil at 1000 rpm for 60 seconds. The PMMA was baked in an oven at 180 °C for 3 minutes. Because the CVD process grew graphene layers on both sides of the copper foil, graphene layers the other side (no PMMA) of the copper foil were removed by oxygen plasma (50 W power for 2 minutes). The copper foil with 50 *μm* thickness was then removed by floating the sample on a copper etchant (Ammonium persulfate 0.1 molar) for 3 hours. To rinse the graphene after copper etching, the copper etchant changed three times with fresh DI water. After the DI water rinsing, PMMA/graphene stack was left on the DI water for 24 hours to remove any impurity and residue of ammonium persulfate and copper oxides. Afterwards, the PMMA/graphene stack was scooped up onto a through-hole SiO_2_/Si chip. Then, the PMMA was removed using acetone and IFM for 2 min followed by thermal annealing at 250 °C for two hours in vacuum (10^−4^ mbar).

### Characterization

Optical microscopy, SEM imaging and optical profilometry (Digital Holographic Microscopy, DHM) were used to observe and characterize the morphology of the devices during and after device fabrication. Confocal Raman spectroscopy (RENISHAW) was used to verify the presence and quality of the monolayer and bilayer graphene. To measure the resonance frequency and quality factor of the structures we used a LDV (OFV-551) from Polytec GmbH with a laser spot size of the order of 2.5 µm to detect the amplitude of the thermomechanical noise of the structures in vacuum (~10^−5^ mbar actively pumped vacuum). Frequency sweeps performed using a lock-in amplifier (UHFLI, Zurich Instruments).
